# ENTWINED LIVES: THE DYADIC RELATIONSHIP BETWEEN CHILDHOOD ADVERSITY AND SOCIAL CONNECTEDNESS AMONG OLDER COUPLES

**DOI:** 10.1093/geroni/igae098.1534

**Published:** 2024-12-31

**Authors:** Yiang Li, Jason Wong, Linda Waite

**Affiliations:** University of Chicago, Chicago, Illinois, United States; Yale University, New Haven, Connecticut, United States; University of Chicago, Chicago, Illinois, United States

## Abstract

Adverse childhood experiences (ACEs) have been linked to disadvantages along the life course. However, little is known about how ACEs may affect one’s own and spousal social connectedness in later life. Using data from the National Social Life, Health, and Aging Project (N=1,214 couples), we employ the actor-partner interdependence model to estimate the long-term dyadic influence of ACE on later-life community participation and social network structure. We find that ACEs (including early-life family unhappiness, absence of parents in the household, poor health, witnessing/experiencing violence, being born in the U.S. South, and low parental education) are associated with reduced later-life community participation and smaller network sizes. Additionally, we find that the spousal spillover effects of ACEs are more pronounced from wives to husbands in fostering social ties than from husbands to wives in engaging with formal community participation. Our findings highlight the spillover effect of childhood disadvantages on spousal later-life social connectedness and its gendered implications.

